# Smart Materials and Devices for Energy Harvesting

**DOI:** 10.3390/ma14164738

**Published:** 2021-08-22

**Authors:** Daniele Davino

**Affiliations:** Department of Engineering, University of Sannio, 82100 Benevento, Italy; davino@unisannio.it

Energy harvesting will be one of the key enabling technologies for the Internet of Things (IoT) world. This technique allows for the powering of wireless sensors and low-power electronics in general, exploiting environmentally available energy. As a matter of fact, the limiting factor for wearable electronics or for wireless sensors in harsh environments is the finite energy stored in the batteries on-board. Indeed, batteries gives a finite duration to the stand-alone performances. The most adopted solution is still to change or recharge the batteries as often as necessary, but this strategy is neither practical, nor economical, nor green-oriented. Indeed, in the case of wireless sensors, located in strategic places in the environment, the replacement, or the recharging of the batteries needs qualified technicians reaching the sensors and doing the operation and this increases the maintenance costs. On the other hand, energy harvesting can convert the energy, right in the place where it is needed. This technique may be exploited also for other applications, such as for the powering of implantable medical/sensing devices for humans and animals.

Energy harvesters based on magnetostrictive alloys are intrinsically robust and long-life. Indeed, these materials inherit most of the mechanical properties of iron, which is the main component of the alloy. The devices make use of kinetic energy and by having no moving parts, are robust and simple because the energy conversion takes place within the material. Indeed, by applying a time variable mechanical stress to the material, a time variable magnetization is obtained (Villari effect) and then a coil can link a variable magnetic flux and produce a voltage. Because of these reasons, they have been proposed for several tasks and even exploited for wearable energy harvesting, with focus on shoes, where high pressures are available because of walking. Piezoelectrics have also been considered for this purpose, but their energy harvesting performance can be reduced after being used several times, due to their brittleness. Fe-Co magnetostrictive alloys can be considered and produced in the form of fibers and integrated into the shoe heels. A relevant energy in the order of µJ can be recovered from a few thousands of steps of usual walking. It seems that the output energy is dependent on user habits of ambulation, not on their weight [1]. On the other hand, exploitation of magnetostrictive harvesters needs careful modeling and simulation because the magneto-mechanical coupling is strongly nonlinear and with hysteresis. A nonlinear model of magnetostrictive materials, such as Galfenol can be developed and its parameters can be determined by using measured magnetostrictives curves. Because of the mechanical, magnetic and electric quantities involved in the device, by using suitable analogies with voltages and currents, a three-port equivalent circuit can be developed. The characterization is performed by applying different compressive force profiles, resistor loads and permanent magnets for the magnetic bias. The modeling and the experiments confirm that the input force frequency and the magnet configuration strongly affect the output voltage and power, while an optimal resistive load, corresponding to the total equivalent coil resistance is needed to extract the maximum power [2]. If more accurate modeling is needed, then a hysteretic Preisach-type model can be considered with a finite element formulation and this approach allows good discrepancies between experimental and computational values of the output power [3].

Piezoelectric devices are the most common energy harvesters converting kinetic energy. Often, they are proposed as a cantilever beam, acting at a specific resonant frequency of the vibrations, or as cylinders directly exposed to the varying force. Since they do not need any coil or bias, they are among the simplest devices and many studies are devoted to the design and the optimization of different geometries to increase conversion [4]. Numerical studies are also useful to determine the best arrangement of the harvester composed of several basic piezo elements, exposed to a varying external force [5] or to model the effect of geometry parameters over the output power in order to get handy formulas [6,7]. Piezoelectrics can also be exploited to directly harvest the energy of variable-speed wind by means of a dynamic multi-stable flutter harvester [8]. Additionally, the search for new materials showing piezoelectricity is active. Electrospun polyvinylidene fluoride (PVDF) fibers can show piezoelectricity but the random arrangement of the fibers, fabricated by traditional electrospinning, reduces the performance and limits the applications. A newly developed 3D electrospinning technique can be exploited to fabricate a PVDF micro wall made up of densely stacked fibers in a fiber-by-fiber manner [9]. This material shows good piezoelectric performance over time and is promising for both energy harvesting and sensing applications.

Large deformations can be harvested by exploiting triboelectricity. Polymers and polymers composites can be arranged as triboelectric nanogenerators where a contact-separation system (10 N of force followed by 5 cm of separation per cycle) can harvest output power ranges from 0.2 to 5.9 mW, depending on the pair of materials, for an active area of 46.4 cm^2^ by using Mica, polyamide (PA66) and styrene/ethylene-butadiene/styrene (SEBS) as positive electrode and polyvinylidene fluoride (PVDF) [10,11], polyurethane (PU), polypropylene (PP) and Kapton as a negative electrode. The highest performance is obtained with Mica with PVDF composites with 30 wt.% of barium titanate (BT) and PA66 with PU pairs.

Temperature gradients are a spread potential source of energy, even if poorly exploitable at a large scale, it is perfectly fitting for energy harvesting purposes. While conventional Thermoelectric Generators (TEGs) are already commercially available, the research frontier on this topic has two main goals: new materials, with improved figure of merits that could be exploited on large surfaces at reasonable costs. For example, a 10-ply glass fiber-reinforced polymer composite laminate can operate as a structural through-thickness TEG. For this purpose, inorganic tellurium nanowires have to be mixed with single-wall carbon nanotubes in a wet chemical approach [12]. This results in a flexible p-type thermoelectric material with a power factor value of 58.88 μW/m·K^2^. Another new device is the thermomagnetic generator (TMG) based on magnetic shape memory alloy (MSMA) films. The TMG generators make use of the concept of resonant self-actuation of a cantilever, caused by a large abrupt temperature-dependent change of magnetization in the material and the rapid heat transfer inherent to the MSMA films [13]. A prototype based on Ni-Mn-Ga film, with a Curie temperature TC of 375 K, has shown a relevant power density of 80 mW/cm^3^ for a heat source temperature of 443 K. This device is modellable with a lumped element model that can be used to estimate the effect of decreasing TC on the lower limit of the heat source temperature in order to predict the possible routes towards waste heat recovery near room temperature [13].

Photovoltaic is the only energy harvesting technique that is scalable from a few mW devices to MW plants. Nevertheless, the challenge is still to reduce the costs by keeping reasonable efficiencies and improving versatility. Thin-film solar cells are one of the solutions. In this framework, several techniques have been proposed and dye-sensitized solar cells (DSSCs) are one of those. The DSSC is relatively simple, semi-flexible and semi-transparent which offers a variety of applications not suitable for glass-based systems and most of the materials used are low-cost. However, few attempts have been tried to further eliminate expensive materials from the process. For example, the use of Fe-modified MgAl-layered double hydroxides (LDHs) to replace dye and semiconductor complexes has shown good results [14], with the MgFeAl-LDH that can act as a simultaneous photoabsorber and charge separator, effectively replacing the dye and semiconductor complex in DSSCs and still yielding an efficiency of 1.56%.

We have discussed several new materials and devices that allow energy harvesting from the environment: magnetostrictives and piezoelectrics, coupling mechanical and/or thermal variables, to electro- or magnetic- variables; materials and devices exploiting temperature gradients for direct conversion into electricity; new materials for more exploitable solar energy conversion and electro-active polymers (EAP) for energy harvesting. These are a few examples but surely there will be many other energy harvesting techniques in the future. Indeed, the field will advance as long as new multi-functional materials will be discovered.

## Data Availability

Not applicable.
